# Enhanced NFκB and AP-1 transcriptional activity associated with antiestrogen resistant breast cancer

**DOI:** 10.1186/1471-2407-7-59

**Published:** 2007-04-03

**Authors:** Yamei Zhou, Christina Yau, Joe W Gray, Karen Chew, Shanaz H Dairkee, Dan H Moore, Urs Eppenberger, Serenella Eppenberger-Castori, Christopher C Benz

**Affiliations:** 1 Buck Institute for Age Research, Novato, CA 94945, USA; 2 Comprehensive Cancer Center, University of California, San Francisco, CA 94143, USA; 3 California Pacific Medical Center Research Institute, San Francisco, CA 94107, USA; 4 Stiftung Tumorbank Basel, Lörracherstrasse 50, Riehen CH-4125, Switzerland

## Abstract

**Background:**

Signaling pathways that converge on two different transcription factor complexes, NFκB and AP-1, have been identified in estrogen receptor (ER)-positive breast cancers resistant to the antiestrogen, tamoxifen.

**Methods:**

Two cell line models of tamoxifen-resistant ER-positive breast cancer, MCF7/HER2 and BT474, showing increased AP-1 and NFκB DNA-binding and transcriptional activities, were studied to compare tamoxifen effects on NFκB and AP-1 regulated reporter genes relative to tamoxifen-sensitive MCF7 cells. The model cell lines were treated with the IKK inhibitor parthenolide (PA) or the proteasome inhibitor bortezomib (PS341), alone and in combination with tamoxifen. Expression microarray data available from 54 UCSF node-negative ER-positive breast cancer cases with known clinical outcome were used to search for potential genes signifying upregulated NFκB and AP-1 transcriptional activity in association with tamoxifen resistance. The association of these genes with patient outcome was further evaluated using node-negative ER-positive breast cancer cases identified from three other published data sets (Rotterdam, n = 209; Amsterdam, n = 68; Basel, n = 108), each having different patient age and adjuvant tamoxifen treatment characteristics.

**Results:**

Doses of parthenolide and bortezomib capable of sensitizing the two endocrine resistant breast cancer models to tamoxifen were capable of suppressing NFκB and AP-1 regulated gene expression in combination with tamoxifen and also increased ER recruitment of the transcriptional co-repressor, NCoR. Transcript profiles from the UCSF breast cancer cases revealed three NFκB and AP-1 upregulated genes – *cyclin D1*, *uPA *and *VEGF *– capable of dichotomizing node-negative ER-positive cases into early and late relapsing subsets despite adjuvant tamoxfien therapy and most prognostic for younger age cases. Across the four independent sets of node-negative ER-positive breast cancer cases (UCSF, Rotterdam, Amsterdam, Basel), high expression of all three NFκB and AP-1 upregulated genes was associated with earliest metastatic relapse.

**Conclusion:**

Altogether, these findings implicate increased NFκB and AP-1 transcriptional responses with tamoxifen resistant breast cancer and early metastatic relapse, especially in younger patients. These findings also suggest that agents capable of preventing NFκB and AP-1 gene activation may prove useful in restoring the endocrine responsiveness of such high-risk ER-positive breast cancers.

## Background

Intracellular responses of ER-positive breast cancers to selective estrogen receptor modulators (SERMs) like tamoxifen are dependent on two different ER-regulated gene mechanisms: one in which liganded ER binds promoter DNA at an estrogen responsive element (ERE), and another in which ER becomes tethered to other promoter-bound transcription factors [1,2]. Nuclear receptors like ER also produce their promoter-regulating effects via ligand-dependent recruitment of co-regulatory factors known as coactivators or corepressors [3-5].

Exhibiting a diverse array of chromatin-modifying activities, coactivators (e.g. SRC1, AIB1/SRC-3, TIF2, CBP/p300, PCAF) mediate the transcription promoting activity of liganded ER whether it is ERE bound or tethered to another promoter-bound complex like AP-1, NFκB, Sp1 or C/EBPβ. Alternatively, the transactivating potential of ER may be repressed by recruitment of a transcriptional corepressor (e.g. NCoR1, SMRT, REA, RIP140) with its associated histone deacetylase activity. The intracellular balance of coactivator-corepressor activity appears to determine, at least in part, whether the net cellular response to tamoxifen-bound ER is agonistic or antagonistic. Much of the antagonistic ER response to tamoxifen is mediated by NCoR1 [6]; and reduced NCoR1 expression in ER-positive primary breast cancers predicts for tamoxifen resistance and early metastatic relapse [7]. However, in several tamoxifen-resistant ER-positive breast cancer models, including those induced by activated ERBB2, a chemical perturbation in the ER DNA-binding domain can reverse tamoxifen resistance by decreasing ER association with AIB1 and increasing its association with NCoR1, without altering cellular expression levels of these two ER co-regulators [8].

Less well appreciated are the intracellular consequences of tamoxifen-liganded ER in association with elevated AP-1 and NFκB transcriptional activities. ER and NFκB are known to be mutually inhibitory at several levels [9]; and it has been suggested that in some ER-positive breast cancers SERMS like tamoxifen can activate NFκB, stimulate cell growth and survival, and thereby contribute to endocrine resistance [10]. Recent clinical evidence suggests that increased NFκB activation, in concert with activated AP-1, identifies a high-risk subset of hormone-dependent breast cancers destined for early relapse on adjuvant tamoxifen therapy [11]. Unlike its interference with NFκB, ER can be recruited by DNA-bound heterodimers from the AP-1 family of b-zip transcription factors; and, dependent on tissue type and balance of nuclear co-regulators, tamoxifen-bound ER that is antagonistic on an ERE-driven gene promoter may be agonistic on an AP-1 driven gene promoter [12-15]. In ER-positive MCF7 cells, upregulated AP-1 activity has been associated with antiestrogen resistance [16]; and in clinical samples of ER-positive breast cancers, tamoxifen resistance has been associated with upregulated AP-1 activity [17].

To model the impact of tamoxifen-liganded ER on NFκB and AP-1 regulated genes, luciferase reporter genes driven by ERE, NFκB or AP-1 were transfected into ER-positive human breast cancer cells shown to possess basal (MCF7) or activated (MCF7/HER2, BT474) NFκB and AP-1 transcriptional activities. NFκB and AP-1 inhibiting doses of parthenolide or bortezomib/PS341, capable of enhancing tamoxifen inhibition of MCF7/HER2 and BT474 growth, were shown to increase recruitment of NCoR1 by endogenous ER in these cells and, relative to MCF7 cells, enhance the antagonistic and inhibitory effects of tamoxifen on both NFκB and AP-1 reporter genes. Finally, to explore the potential of linking genes upregulated by both NFκB and AP-1 with tamoxifen resistance, expression microarray data available from 54 node-negative ER-positive breast cancers revealed three such genes capable of dichotomizing these cases into early and late relapsing subsets despite adjuvant tamoxifen therapy: *cyclin D1*, *uPA *and *VEGF*. The prognostic association of these genes with patient outcome was further evaluated using node-negative ER-positive breast cancer cases identified from three other published data sets (Rotterdam, n = 209; Amsterdam, n = 68; Basel, n = 108), each having different patient age and adjuvant tamoxifen treatment characteristics.

## Methods

### Reagents, breast cancer cell lines, and drug response interactions

Unless otherwise specified, all reagents were purchased from Sigma Chemical Co. (St. Louis, MO), including tamoxifen (TAM; [Z]-1- [p-dimethylaminoethoxyphenyl]-1,2-diphenyl -1 butene). Parthenolide (PA) was purchased from Alexis Biochemicals (San Diego, CA); and bortezomib (PS341) was kindly provided by Millennium Pharmaceuticals Inc. (Cambridge, MA). The ER-positive/ERBB2-negative MCF7 and ER-positive/ERBB2-positive BT474 human breast cancer cell lines were obtained from the American Type Culture Collection (Rockville, MD) and were maintained at 37°C and 5% CO_2 _in Dulbecco's modified Eagle's medium (DMEM) for MCF7 or RPMI-1640 medium for BT474, supplemented with 10% fetal bovine serum (FBS), 1% penicillin-streptomycin, and 10 ng/ml insulin. Media and supplements were purchased from Mediatech, Inc. (Herndon, VA). The ER-positive/ERBB2-positive subline, MCF7/HER2 (clone-18), was maintained like parental MCF7 cells except for selection under G418 as originally reported [18]. Data from previously reported experiments were re-analyzed to determine TAM-PA and TAM-PS341 treatment interactions [11,19]. Relative to vehicle (DMSO or ethanol) treated controls, single agent and combined treatments (at indicated dose and duration) were assessed after plating 10^4 ^cells (MCF7, MCF7/HER2, BT474) in 24-well microtiter dishes by sulforhodamine B (SRB) viability assay. Expected (E) degrees of growth inhibition following TAM-PA or TAM-PS341 treatment combinations were determined based on independent probabilities of proportional growth reduction, and these were compared with observed (O) degrees of growth reduction (control = 1.0 or 100%). Following replicate testing (three independent experiments in quadruplicate), mean ± SEM O/E values were calculated and the significance (p-values) for O/E reductions in MCF7/HER2 or BT474 relative to MCF7 cells were determined by ANOVA and *t *testing.

### Immunoprecipitation, immunoblotting and DNA-binding assays

Whole cell lysates were used for immunoprecipitation and immunoblotting. Cells were extracted in NP-40 buffer containing 50 mM Hepes (pH 7.4), 150 mM NaCl, 1% Nonidet P-40, 25 μM β-glycerol phosphate, 25 mM sodium fluoride, 5 mM EGTA, 1 mM EDTA, 1 mM sodium vanadate, and a cocktail of mini-complete protease inhibitors (Roche Diagnostics, Mannheim, Germany). Protein aliquots of 15–30 μg were separated by gel electrophoresis in 4–12 % Bis-Tris SDS gradient gels in morpholinepropane sulfonic acid (MOPS) running buffer. Proteins transferred onto polyvinylidene difluoride (PVDF) membranes were first blocked (5% non-fat milk in PBS containing 0.1% Tween-20) and then incubated overnight with antibodies specific to ERα, p50, p65, or bcl-3 (Santa Cruz Biotechnology Inc., Santa Cruz, CA). Immunoreactive bands were visualized with horseradish peroxidase-conjugated goat-anti-mouse IgG (BioRad, Hercules, CA) and chemiluminescence enhancement reagents (Pierce, Rockford, IL). Membranes were then stripped and reblotted with β-actin antibody (Abcam Inc., Cambridge, MA) to normalize for protein loading. For immunoprecipitation (IP) followed by Western blotting (WB), 300 μg protein aliquots were first incubated with 10 μl of p50 or NCoR antibody solution (Santa Cruz Biotechnology Inc., Santa Cruz, CA) for 2 hours (4°C) under continuous agitation. Immune complexes were recovered by adding 25 μl of Protein A-Sepharose beads (Amersham Biosciences, Uppsala, Sweden), washing × 5 in lysis buffer, and resuspending in 3 × Laemmli sample buffer prior to gel electrophoresis and immunoblotting, as described above.

### Transient transfection and luciferase reporter assays

Cultures were seeded one day before transfection with luciferase (luc) reporters to a density of 1–2 × 10^3 ^cells per well in 96-well microtiter plates and using appropriate growth media. Cells were transiently transfected with either 0.5 μg of (ERE)_3_-tk-luc reporter plasmid (Promega, Madison, MI) or 0.5 μg of (NFκB)-luc or (AP-1)-luc reporter plasmid (Stratagene, La Jolla, CA), along with FuGene 6 transfection reagent (Roche, Indianapolis, IN). The *Renilla *luciferase vector pRL-tk-luc (Promega, Madison, MI) was co-transfected to normalize for transfection efficiency. Culture media was changed 20 h following transfection and cells were then treated with PS341 (10 nM, 25 nM) or PA (1 μM, 5 μM), alone or in combination with 500 nM TAM for 24 h. Cells were subsequently washed with PBS, lysed for Dual-Glo™ luciferase assay (Promega), and reporter activity measured by luminometer. The ratio of firefly luminescence/*Renilla *luminescence was used for comparison of AP-1 and NFκB driven gene activities in the untreated cell lines. All other transfections were reported as fold-changes in luciferase activity over vehicle treated controls. All transient transfection and reporter gene results are presented as mean values (± SEM) from at least three independent experiments using quadruplicate conditions. MCF7/HER2 and BT474 results showing significant differences (p < 0.05) from comparably treated MCF7 controls were determined by one-way ANOVA and *t *testing.

### Breast cancer datasets and genes upregulated by NFκB and AP-1

Cryobanked breast cancer specimens were obtained from the University of California San Francisco (UCSF) Comprehensive Cancer Center Breast Oncology Program Tissue Core, and collected under UCSF approved protocols following patient consent. From an archive of over 1,000 liquid nitrogen frozen breast cancer specimens, 54 primary breast cancer samples (UCSF cases) had been identified for other study purposes [20], using the following criteria: early clinical stage (T1/2, N0, M0) invasive breast cancer, ER-positive status (>10% nuclear immunohistochemical staining), known primary systemic therapy (treatment era: 1989–2004, with 33/54 receiving adjuvant tamoxifen and 18/54 receiving adjuvant chemotherapy), known clinical outcome (relapse-free survival, RFS), and stratification into young (≤ 45 years, n = 29) or old (≥ 70 years, n = 25) age-at-diagnosis. The age stratification groups were balanced for standard prognostic markers (tumor size, grade, proliferation index, ERBB2 status) and adjuvant therapy, but showed differences in clinical outcome with the younger cases experiencing more frequent (7/29) and earlier metastatic relapses (median RFS = 4.84 years, range 0.13–14.14 years) than the older age-at-diagnosis cases (2/25; median RFS = 5.52 years, range 0.74–12.72 years). Sample requirements also included a minimum frozen-wet weight of 100 mg and histologic confirmation of >50% cancer cells per sample section. Total RNA from each tissue sample was formamide extracted, purified and reconstituted in aqueous solution using RNeasy (Qiagen, CA) according to manufacturer's instructions; and the resulting RNA samples were quality verified by micro-analysis (Agilent Bioanalyzer, CA). Total RNA (3–5 μg per sample) was labeled and analyzed using Affymetrix (Santa Clara, CA) high-density oligonucleotide microarrays, HG-U133A (v2), in 96-well chips each with 22.2 K annotated probes representing ~13 K unique Unigenes. Gene expression analysis was performed using standard Affymetrix procedures within the Lawrence Berkeley National Lab and Life Science Division's Molecular Profiling Laboratory (MPL). Probe set measurements were generated from quantified Affymetrix image files (.CEL files) using the RMA algorithm from BioConductor R. The resulting data matrix consisted of normalized log abundance values for each probe set and sample; these data files and associated clinical parameters for each sample have been entered into the NCBI Gene Expression Omnibus (GEO) repository (GSE7378). Gene expression values were mean centered, and a low variation filter was applied to exclude probe sets that did not have at least 5 observations exhibiting a two-fold change from the mean. While arbitrary, this filter restriction was based on accepted criteria [21]. Filtered probes were annotated by GeneTraffic software (MPL) and those with unknown Unigene identity were omitted, yielding a final significant set of 5,523 probes representing 4,332 unique genes. This significant probe set was searched for genes reported to be coordinately upregulated by both NFκB and AP-1 [22-26]. To dichotomize the 54 tumor samples according to levels of expression for each of the NFκB and AP-1 upregulated genes identified in the significant probe set, samples with positive mean-centered transcript values were designated as "high" expressors while those with negative mean-centered values were classified as "low" expressors. Kaplan-Meier RFS curves were produced for each of the high/low dichotomizations, and Log Rank analysis was used to determine statistical significance between survival curves (StatView, SAS Institute Inc).

Three other independent data sets were used to evaluate the prognostic value of the NFκB and AP-1 upregulated genes identified from the UCSF dataset. Node-negative ER-positive breast cancer cases were identified from published Amsterdam (n = 68), Rotterdam (n = 209) and Basel (n = 108) gene expression studies, each consisting of cases with different age and adjuvant tamoxifen treatment characteristics (21, 27, 28). The Amsterdam dataset was obtained through the Rosetta Inpharmatics publications archive and consisted of breast cancers diagnosed in younger age (< 55 years) patients, of which 95% received no adjuvant therapy [21]; corresponding gene probes were identified by UniGene symbols. The Rotterdam dataset was obtained through the NCBI/Genbank GEO database (GSE2034) and consisted of breast cancers diagnosed in patients with ages ranging from 26–83 years, none of whom received any adjuvant therapy [27]; corresponding gene probes were identified by Affymetrix identification numbers, and when a gene was represented by multiple probes, the average expression value was calculated. The node-negative ER-positive Basel dataset was extracted from a previously reported study population, and consisted of breast cancers diagnosed in patients with ages ranging from 37–84 years, the majority of whom (70%) received adjuvant tamoxifen therapy [28]. For the Amsterdam dataset, gene expression levels were determined using an Agilent microarray platform; positive log10(ratio) values were designated as "high" expressors, while those with negative log10(ratio) values were designated as "low" expressors. For the Rotterdam dataset, gene expression levels were determined using an Affymetrix microarray platform; positive mean centered values were designated as "high" expressors and negative mean centered values were designated as "low" expressors. For the Basel dataset, a median cut-point was applied to the quantitative real-time PCR data to dichotomize the samples into "high" vs. "low" expressors. Kaplan-Meier metastasis free survival curves were produced for each of the high/low dichotomizations, and Log Rank analysis was used to determine statistical significance between survival curves.

## Results

### Tamoxifen resistant breast cancer models show enhanced NFκB and AP-1 transcriptional activity

**Figure 1:**
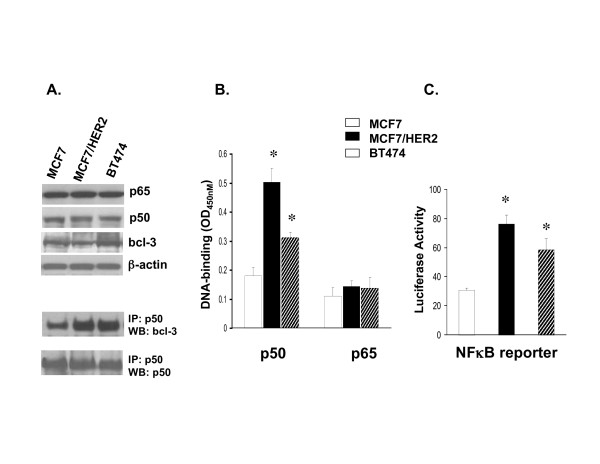
Increased DNA-binding and transcriptional activity of NFκB complexes expressed in TAM-resistant (MCF7/HER2, BT474) relative to TAM-sensitive (MCF7) breast cancer cells. **A. **Whole cell lysates were immunoblotted to compare endogenous protein expression of p50, p65, and bcl-3 components relative to β-actin. Aliquots were also immunoprecipitated (IP) with antibody to p50, and subsequently Western blotted (WB) to detect bcl-3 complexed to p50 relative to total immunoprecipitated p50. **B. **DNA-binding by individual NFκB p50 and p65 subunits from nuclear extracts, quantitated by ELISA-based Trans-AM™ assays (mean ± SE OD_450nm _values from triplicate samples). **C. **(NFκB)-luciferase reporter gene expression (mean ± SE values from triplicate samples) measured after transient co-transfection of cells with the firefly reporter plasmid and a *Renilla *luciferase vector for normalization, as described in Methods. Asterisks (*) indicate significant difference (p < 0.05) from control MCF7 values.

**Figure 2:**
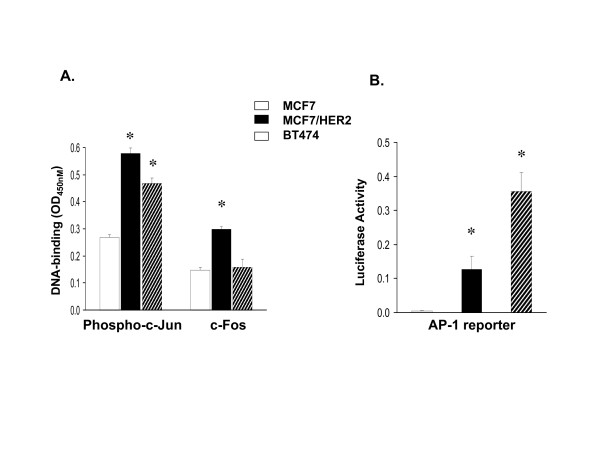
Increased DNA-binding and transcriptional activity of AP-1 complexes expressed in TAM-resistant (MCF7/HER2, BT474) relative to TAM-sensitive (MCF7) breast cancer cells. **A. **DNA-binding by individual phospho-c-Jun and c-Fos subunits from nuclear extracts, quantitated by ELISA-based Trans-AM™ assays (mean ± SE OD_450 nm _values from replicate samples). **B. **(AP-1)-luciferase reporter gene expression (mean ± SE values from triplicate samples) measured after transient co-transfection of cells with the firefly reporter plasmid and a *Renilla *luciferase vector for normalization, as described in Methods. Asterisks (*) indicate significant difference (p < 0.05) from control MCF7 values.

To study the effects of NFκB and AP-1 upregulated gene expression in ER-positive breast cancer cells, two TAM-resistant ER-positive/ERBB2-positive breast cancer cell lines, MCF7/HER2 and BT474, were evaluated in relation to TAM-sensitive ER-positive/ERBB2-negative MCF7 cells. Figure 1 demonstrates the 2-to-3 fold increased levels of NFκB DNA-binding and NFκB driven reporter gene (luciferase) expression in both MCF7/HER2 and BT474 compared to MCF7 (panels B and C, respectively), despite comparable levels of NFκB p65 and p50 component proteins in all three cell lines (panel A). Since the increased NFκB activation in both MCF7/HER2 and BT474 appeared largely due to increased p50 DNA-binding (panel B), and NFκB p50 is known to lack a transactivation domain and require Bcl-3 binding to become transcriptionally competent [29-31], IP-immunoblotting was used to demonstrate that the p50 extracted from BT474 and MCF7/HER2 were associated with 3-to-4 fold more Bcl-3 than the p50 extracted from MCF7 cells (panel A). Similarly, Figure 2 demonstrates that while MCF7/HER2 and BT474 exhibited only about 2-fold greater AP-1 DNA-binding over MCF7, largely composed of phospho-c-Jun (panel A), these cell lines showed at least 10-to-30 fold greater levels of AP-1 driven reporter gene activity (panel B). Altogether, Figure 1 and 2 findings confirm that the ER-positive/ERBB2-positive MCF7/HER2 and BT474 cell lines contain significantly greater levels of NFκB and AP-1 activity relative to the ER-positive/ERBB2-negative MCF7 cells.

### Enhanced tamoxifen responsiveness associated with suppression of NFκB and AP-1 regulated gene expression

Previous studies have adequately demonstrated that ER-positive/ERBB2-positive breast cancer models simulate clinical cases by exhibiting TAM-resistance relative to TAM-sensitive ER-positive/ERBB2-negative models and cases [18,32,33]. These same TAM-resistant ER-positive/ERBB2-positive models have been used to demonstrate that NFκB inhibiting doses of the IKK inhibitor, parthenolide (PA), or the proteasome inhibitor, bortezomib (PS341), can be used to significantly improve sensitivity to TAM [11]. Data from these previous experiments [11] were re-analyzed to quantitate TAM-PA and TAM-PS341 treatment interactions in MCF7/HER2 and BT474 cells relative to MCF7, as shown in the O/E values presented in Table 1. BT474 cells showed somewhat greater synergistic TAM-PA and TAM-PS341 interactions relative to MCF7HER2 cells for the two PA (1, 5 μM) and PS341 (10, 25 nM) doses; still, the greater than additive interactions observed in TAM-resistant MCF7/HER2 relative to the TAM-sensitive MCF7 resulted in comparable final growth inhibitory effects in both cell lines treated with these TAM combinations. To understand the basis for these synergistic TAM-PA and TAM-PS341 growth inhibitory responses, gene reporter studies were performed in cell cultures treated for 24 h with the same drug doses and combinations shown in Table 1.

**Figure 3:**
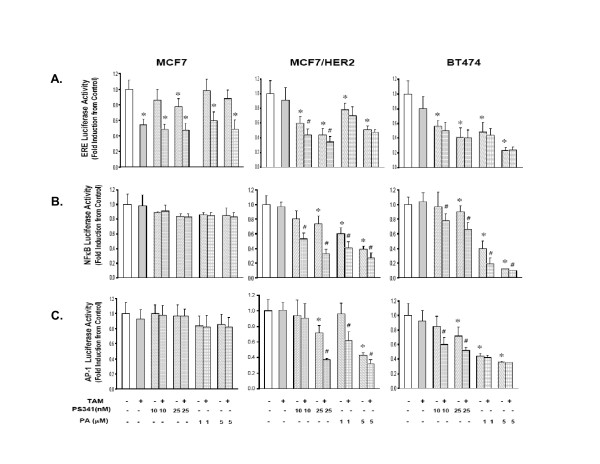
Comparison of drug treatment effects on expression of (ERE)-luciferase (**A**), (NFκB)-luciferase (**B**), and (AP-1)-luciferase (**C**) reporter genes in TAM-resistant (MCF7/HER2, BT474) and TAM-sensitive (MCF7) breast cancer cells. As described in Methods, cell cultures were transiently transfected with the specific reporter plasmid 20 h before a 24 h culture treatment with the indicated dose of either PS341 or PA, alone or in combination with TAM (500 nM). Reporter activity (firefly luciferase) is presented as mean ± SE fold induction over vehicle treated control cells. * p < 0.05, for drug alone vs. vehicle control; # p < 0.05, for TAM-PS341 or TAM-PA combinations vs. PS341 or PA alone.

Treatment for 24 h with either PA (1, 5 μM) or PS341 (10, 25 nM), alone or in combination with TAM (500 nM), did not affect protein expression of ERα, PR_A _or PR_B _(data not shown) in any of the cell lines. The gene-regulating effects of PA and PS341, alone and in combination with TAM, were evaluated in MCF7, MCF7/HER2 and BT474 cells transiently transfected with luciferase (luc) reporter constructs driven by either ERE, NFκB or AP-1 regulated promoters. Figure 3A shows that TAM alone significantly inhibited (ERE)-luc activity in TAM-sensitive MCF-7 cells but not in TAM-resistant MCF7/HER2 or BT474 cells. In contrast, as single agents PA and PS341 inhibited (ERE)-luc activity in MCF7/HER2 and BT474 cells but not in MCF7 cells; and TAM combinations with either PA or PS341 produced no significant further reductions in (ERE)-luc activity over that produced by PA or PS341 alone. Unlike the treatment effects on (ERE)-luc expression in all three cell lines, NFκB and AP-1 driven reporters showed no treatment responses in TAM-sensitive MCF7 cells. Following the same drug dosing associated with synergistic TAM-PA and TAM-PS341 growth inhibitory responses in MCF7/HER2 and BT474 cells (Table 1), Figure 3B shows synergistic suppression of (NFκB)-luc expression only in the NFκB overexpressing TAM-resistant models. Likewise, and with the single exception of TAM-PA treatment in BT474 cells, Figure 3C shows significant synergistic suppression of (AP-1)-luc expression only in AP-1 overexpressing TAM-resistant models.

### Enhanced tamoxifen responsiveness associated with ER recruitment of the co-repressor, NCoR

**Figure 4:**
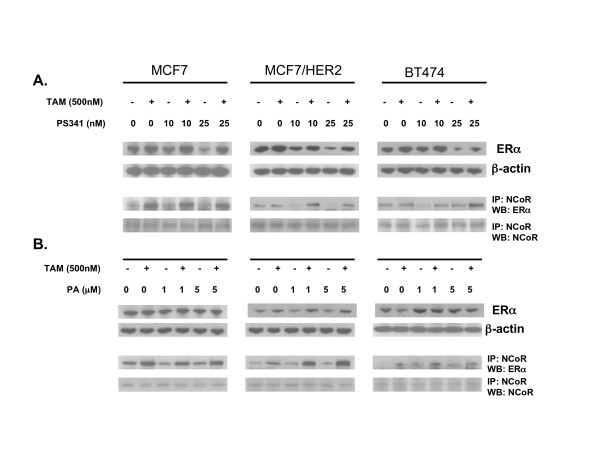
Comparison of drug treatment effects on ER and ER binding to the transcriptional co-repressor, NCoR, in TAM-resistant (MCF7/HER2, BT474) and TAM-sensitive (MCF7) breast cancer cells. Cell cultures were treated for 24 h with vehicle, PS341 (panel **A**), or PA (panel **B**), alone or in combination with TAM (500 nM). Whole cells were then extracted and Western blotted to detect total ER relative to β-actin, or immunoprecipitated (IP) and then Western blotted (WB) to detect NCoR-bound ER relative to total immunoprecipitated NCoR.

TAM-PA and TAM-PS341 drug combinations associated with synergistic growth inhibition and reductions in NFκB and AP-1 driven reporter gene expression were also evaluated with regard to ER recruitment of the transcriptional co-repressor, NCoR. As shown in the IP-immunoblots of Figure 4, neither PA nor PA341, administered as single agents, produced any significant change in MCF7, MCF7/HER2 or BT474 levels of co-repressor bound to ER. As would be expected in TAM-sensitive cells, within 24 h of TAM treatment MCF7 showed a >3 fold increase in the amount of NCoR-bound ER. In contrast, TAM-resistant MCF7/HER2 and BT474 showed little if any TAM induced change in NCoR-bound ER. After combination treatment with either TAM-PA or TAM-PS341, MCF7/HER2 and BT474 showed levels of NCoR-bound ER approaching that seen in treated MCF7 cells.

### Analysis of ER-positive primary breast cancers for NFκB and AP-1 upregulated genes

Given the fact that our TAM-resistant breast cancer models, MCF7/HER2 and BT474, showed activation of both NFκB and AP-1 transcription factor complexes by both DNA-binding and reporter gene assays, we performed an exploratory analysis in a UCSF dataset of 54 ER-positive, node-negative primary human breast cancers looking for prognostic genes known to be upregulated by activated NFκB and AP-1. Using a commercial expression microarray platform and standard filtering criteria, 4,332 unique genes were found to be variably expressed within this set of histologically similar, early-stage ER-positive breast cancers. Interestingly, the transcript levels for many NFκB constituents, including *p50/NFKB1*, *p60*, *p65/RelA*, *RelB*, and *NFKB1A/IKBα *did not pass a commonly used low variation expression threshold. Only one NFκB related probe (*c-Rel*) showed sufficient expression variation to be included in the significant probe set. In contrast, six AP-1 components passed the low variation expression filter, including *c-Jun*, *JunB*, *JunD*, *c-Fos*, *FosB*, and *Fra2*. Except for *JunD*, none of these variably expressed NFκB or AP-1 components showed any significant association with patient outcome. Among NFκB and AP-1 regulated genes showing sufficient expression variation were three probes for *VEGF*, two probes each for *uPA *and *cyclin D1*, and one probe each for *MMP9*, *CCL2*, ICAM1, *AGT *and *BF*. Several other well known NFκB regulated genes (e.g. *COX2*, *TNF*, *LTA*, *CSF2*, *CSF3*, *IFNB1*, *IFNG, IL-6, IL-8*) failed to pass the low variation filtering criteria for admission into the significant probe set expressed by these early-stage ER-positive breast cancers.

**Figure 5:**
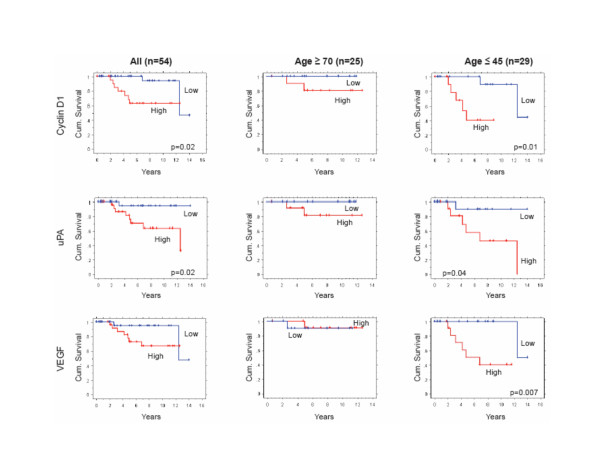
Kaplan-Meier relapse-free survival of 54 UCSF node-negative, ER-positive breast cancer cases by age cohort (≥ 70, ≤ 45 years) and with outcomes dichotomized for tumor expression (high, low) of the NFκB and AP-1 regulated genes *cyclin D1*, *uPA*, and *VEGF*. As described in Methods, the 54 node-negative cases were balanced between cohorts for standard prognostic markers and adjuvant TAM treatment (>60%). Gene transcript levels were quantified using a commercial microarray platform with standard data processing (Affymetrix); cases were designated high or low expressors based on mean-centered gene expression. Significant differences between the cumulative survival curves were determined by Log Rank analyses (only p values < 0.05 shown)

Microarray determined "high" vs. "low" transcript levels for the eight NFκB and AP-1 regulated genes variably expressed in the 54 UCSF breast cancer cases were evaluated in relation to patient relapse-free survival (RFS), adjusted for prior adjuvant tamoxifen use and patient age-at-diagnosis. Of these, only *VEGF*, *uPA *and *cyclin D1 *showed significant outcome associations with RFS as shown in the Kaplan-Meier plots of Figure 5, indicating that high expressors for each of the three genes represented cases most likely to relapse. Of interest, the prognostic value of these three NFκB and AP-1 regulated genes measured by separation between RFS curves, appeared greater for the younger age cases (≤ age 45 years) in which metastatic relapses occurred earlier and more frequently relative to the older age cases (≥ 70 years), despite the fact that tumors from both age cohorts were indistinguishable with regard to standard prognostic tumor markers, use of systemic adjuvant therapy, and level of expression for each of these genes. Over 60% of these cases were treated with adjuvant tamoxifen, and since recurrences among both older and younger cases occurred independent of adjuvant tamoxifen and usually within 5 years of diagnosis, relapsing breast cancers were considered tamoxifen resistant. All 54 cases could be designated as either high (H) or low (L) expressors for each of the three NFκB and AP-1 regulated genes and, thus, combinatorially subdivided into four categories (HHH, n = 10; HHL, n = 16; HLL, n = 14; LLL, n = 14). Kaplan-Meier analyses demonstrated significantly worse RFS according to the number of high expressing NFκB and AP-1 regulated genes (Log Rank p = 0.03), with those cases showing high expression of all three genes (HHH) exhibiting a median RFS of < 5 years (data not shown).

**Figure 6:**
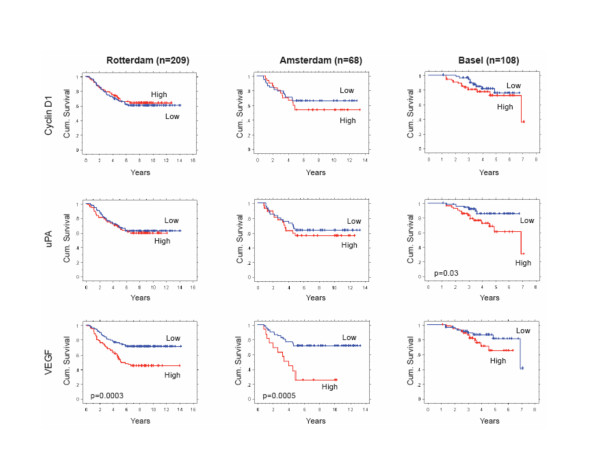
Kaplan-Meier metastasis-free survival of node-negative ER-positive breast cancer cases identified from three independent data sets (Rotterdam, Amsterdam, Basel), with outcomes dichotomized for tumor expression (high, low) of the NFκB and AP-1 regulated genes *cyclin D1*, *uPA*, and *VEGF*. As described in Methods, the Rotterdam cases (n = 209) represented patients of all ages (26–83 years), none of whom received any adjuvant therapy; gene transcripts were measured by commercial (Affymetrix) microarray platform, and high/low dichotomization based on mean centered expression levels. The Amsterdam cases (n = 68) represented young patients (all < 55 years), only 5% of whom received adjuvant TAM; gene transcripts were measured by commercial (Agilent) microarray platform, and high/low dichotomization based on positive/negative log10 gene expression ratios. The Basel cases (n = 108) represented patients of all ages (37–84 years), 70% of whom received adjuvant TAM therapy; gene transcripts were measured by quantitative real-time reverse transcriptase polymerase chain reaction (qRT-PCR) assay, and high/low dichotomization based on median cut-point gene transcript levels. Significant differences between the cumulative survival curves were determined by Log Rank analyses (only p values < 0.05 shown).

As shown in the Kaplan-Meier plots of Figure 6, the prognostic associations of these three NFκB and AP-1 upregulated genes were further evaluated in node-negative ER-positive breast cancer cases identified from three other published data sets (Rotterdam, n = 209; Amsterdam, n = 68; Basel, n = 108), each with different patient age and adjuvant tamoxifen treatment characteristics and none fully consistent with the UCSF characteristics (21, 27, 28). Across all three datasets, higher levels of the NFκB and AP-1 upregulated genes were generally associated with worse outcome although the outcome differences between the high/low dichotomized cases often did not reach statistical significance (p < 0.05). In the older age, tamoxifen-untreated Rotterdam cases and in the younger age, tamoxifen-untreated Amsterdam cases only *VEGF *showed significant prognostic impact; in the older age, tamoxifen-treated Basel cases only *uPA *showed significant prognostic impact. Consistent with the UCSF results, when all three NFκB and AP-1 upregulated genes were combined to produce four subsets within each dataset, the resulting Kaplan-Meier curves showed a rank order correlation between increasingly worse outcome and number of high expressing genes (HHH > HHL > HLL > LLL); although these outcome differences did not quite reach statistical significance by Log Rank analysis, in each dataset those cases showing high expression of all three NFκB and AP-1 upregulated genes (HHH) exhibited median times to metastatic recurrence ≤ 5 years (data not shown).

## Discussion

A number of gene signature studies have now demonstrated that ER-positive breast cancers can be subset into those associated with good patient prognosis and those at higher risk for metastatic relapse despite adjuvant endocrine therapy [32,34-36]. Other preclinical and clinical evidence links TAM-resistant ER-positive breast cancer with co-expression of one or more members of the ERBB/HER family of membrane receptor tyrosine kinesis [32,33,37-40], activation of insulin-like growth factor receptors [41], and/or constitutively active downstream signal transduction pathways (e.g. Ras/Raf, PKC, MAPK, PI3K/AKT/mTOR) capable of crosstalking with ER [38,42-45]. These ER interfering signals all converge on two transcription factor complexes, AP-1 and NFκB, also found activated in high-risk subsets of ER-positive breast cancer relapsing early on TAM therapy [17,19]. A recent analysis of 59 early stage ER-positive breast cancers, diagnosed across all age groups and treated with adjuvant TAM, identified increases in both NFκB (p50) and AP-1 DNA-binding as co-predictors of early metastatic relapse [19]. Cross correlation of these two parameters with numerous other protein biomarkers formerly evaluated in the same tumor set also identified a strong association with *uPA *expression [19], an established prognostic marker for node-negative breast cancer [28] transcriptionally controlled by the cooperative transactivation of both NFκB and AP-1 [23]. Thus, this study introduced the possibility that ER-positive breast cancers at risk for antiestrogen resistance and early metastatic relapse might be identified at diagnosis by elevated expression of genes transcriptionally upregulated by NFκB and AP-1. Still to be defined, however, was the mechanistic rationale for a therapeutic intervention that could prevent the activation of NFκB and AP-1 and thereby restore antiestrogen sensitivity to such high-risk ER-positive breast cancers.

In the present study, two TAM-resistant ER-positive/ERBB2-positive breast cancer cell lines (MCF7/HER2 and BT474) and one TAM-sensitive ER-positive/ERBB2-negative breast cancer cell line (MCF7) were used to model the effects of TAM treatment on breast cancers with either low or high levels of NFκB and AP-1 transcriptional activity, and assess the TAM interacting effects of drugs (PA, PS341) potentially capable of lowering NFκB and AP-1 activities. These cell lines are considered relevant experimental models since TAM acts as a growth antagonist in MCF7 but shows little growth inhibiting activity against BT474 and can actually stimulate growth of MCF7/HER2 [18], similar to clinical observations with TAM treated ER-positive/ErbB2-positive breast cancers [33]. While the TAM-resistance displayed by MCF7/HER2 and BT474 undoubtedly results from their constitutively activated ERBB2 signaling, the present studies demonstrated that these models exhibit significantly increased NFκB (Figure 1) and AP-1 (Figure 2) DNA-binding and gene upregulating activities. Unlike observations with MCF7 cells, TAM treatment failed to significantly suppress (ERE)-luc reporter gene activity in MCF7/HER2 and BT474 cells (Figure 3A), and produced only attenuated recruitment of the co-repressor NCoR to TAM-bound ER (Figure 4). Earlier studies comparing parental MCF7 with MCF7/HER2 had demonstrated that both cell lines express comparable levels of NCoR, and that TAM treatment of MCF7/HER2 fails to result in NCoR recruitment by ER but inhibition of ERBB2 signaling can restore ER-NCoR binding in MCF7/HER2 compareble to that seen in MCF7 cells [46].

Rather than inhibit upstream ERBB2 signaling, the present approach was designed to inhibit downstream NFκB and AP-1 activation in the TAM-resistant breast cancer models. PA and PS341, at doses capable of enhancing TAM induced growth inhibition of MCF7/HER2 and BT474 cells (Table 1), reduced NFκB and AP-1 driven gene expression (Figures 3B, C); and, in combination with a TAM dose that alone had no effect on these reporter genes, TAM-PA and TAM-PS341 combinations more substantially reduced NFκB and AP-1 driven gene expression but only in the TAM-resistant cells having constitutively elevated NFκB and AP-1 activity. These same TAM-PA and TAM-PS341 combinations were able to restore the levels of NCoR binding to TAM-liganded ER in the MCF7/HER2 and BT474 cells toward that observed in the TAM-sensitive MCF7 cells. A more general extrapolation of these results would suggest that direct inhibition of constitutively elevated NFκB and AP-1 transcriptional activities, caused by any number of oncogenic stimuli and signaling pathways, can improve the endocrine responsiveness of otherwise TAM-resistant ER-positive breast cancers by enhancing NCoR recruitment to TAM-liganded ER, thereby suppressing expression of growth and metastasis promoting genes upregulated by both NFκB and AP-1.

Looking for preliminary evidence of growth and metastasis promoting genes coordinately upregulated by both NFκB and AP-1, expression microarray data available from 54 UCSF node-negative ER-positive breast cancers with known clinical outcome revealed three such genes capable of dichotomizing these cases into early and late relapsing subsets despite adjuvant tamoxifen therapy: *cyclin D1*, *uPA *and *VEGF *(Figure 5). Not only are these gene candidates known to be transcriptionally upregulated by the coordinated activation of both NFκB and AP-1 [22-26], their overexpression has been linked to clinically aggressive forms of breast cancer and, in some cases, to tamoxifen resistance [28,47,48]. For unrelated study purposes [20], these ER-positive tumor samples had been preselected from two different age-at-onset breast cancer case populations (young, ≤ age 45; older, ≥ 70), but were otherwise indistinguishable with regard to standard prognostic markers and systemic adjuvant therapy including TAM. While overexpression of these three NFκB and AP-1 upregulated genes at the protein level has been well documented for clinically aggressive breast cancers, their prognostic impact at the transcript level had not been previously recognized for ER-positive breast cancer. Of interest, the prognostic value of these genes measured by separation in Kaplan-Meier RFS curves appeared greater for the younger age-at-onset cases, in which there were overall more metastatic relapses than for the older age-at-onset cases, despite the fact that both age groups showed similar median and range transcript values for each of the gene candidates. Since higher expression of more than one of these three genes might be expected given a common NFκB and AP-1 upregulating transcriptional mechanism, it is of interest that the 26 cases showing 2/3 (HHL) or 3/3 (HHH) of these upregulated genes showed significantly worse RFS outcomes than the 28 cases with fewer upregulated genes (HLL, LLL), and that the 10 early stage ER-positive breast cancers with all three upregulated genes had the poorest outcome with a median RFS of < 5 years.

The associations of *cyclin D1*, *uPA *and *VEGF *with patient outcome were tested in node-negative ER-positive breast cancer cases identified from three other independent datasets (21, 27, 28), each with different patient age and adjuvant tamoxifen treatment characteristics and none comparable to the younger age, tamoxifen-treated UCSF cases in which these three genes exhibited their greatest prognostic impact. Identified from these other datasets were 209 older age, tamoxifen-untreated Rotterdam cases, 68 younger age, tamoxifen-untreated Amsterdam cases, and 108 older age, tamoxifen-treated Basel cases. Consistent with the UCSF results, higher expression levels of these three NFκB and AP-1 upregulated genes in the other datasets were generally associated with worse outcomes, individually and collectively, although many of these differences did not quite reach statistical significance. It is important to note that no effort was made to optimize the high/low median value cut-points arbitrarily chosen for the UCSF outcome analysis and subsequently applied to the other datasets. Generalizing from all four datasets it might be suggested that as individual prognostic factors, VEGF appeared least dependent on age-at-diagnosis and adjuvant tamoxifen status, uPA appeared most useful in tamoxifen treated cases, and the impact of cyclin D1 appeared limited to younger cases. Evaluating the three NFκB and AP-1 upregulated genes collectively across all four datasets and weighting them equally demonstrated that node-negative ER-positive cases with high level expression of all three genes (HHH) experience the earliest metastatic relapses (median recurrence intervals ≤ 5 years).

While exploratory and preliminary in nature, these transcript expression results indicate that genes coordinately upregulated by NFκB and AP-1 may be implicated in the clinical behavior of ER-positive breast cancers resistant to tamoxifen and destined for early relapse. Since the transcriptional activities of NFκB and AP-1 complexes are not directly measurable in clinical breast cancer samples, prognostic signatures reflecting these activities would help to identify high-risk ER-positive breast cancers in need of more aggressive adjuvant therapy. Although *cyclin D1*, *uPA *and *VEGF *are known to be transcriptionally upregulated by NFκB and AP-1, the promoters of these three genes are also regulated by numerous other transcription factors which no doubt possess variable activities in otherwise similar ER-positive node-negative breast cancers, likely contributing to the variable prognostic impact of these genes observed across the four independent datasets. Based on the outcome associations demonstrated here for *uPA*, *cyclin D1*, and *VEGF *transcript levels as extracted from frozen breast cancer samples and quantitated by different assay platforms, future studies might consider validating this minimal gene signature set using RNA extracted from formalin-fixed and paraffin-embedded breast cancer samples, much like the recently approved OncoType DX^® ^(Genomic Health) assay which employs a 21-gene signature set to identify ER-positive node-negative breast cancers in need of aggressive adjuvant therapy [49]. As well, a validated gene signature set representing constitutively activated NFκB and AP-1 activity in ER-positive breast cancers might be used to identify patients who would be optimally treated with an antiestrogen in combination with an NFκB and AP-1 down-regulating drug [11,50]. Such drugs might include a proteasome inhibitor (e.g. bortezomib, NPI-0052), an IKK inhibitor (e.g. BAY11-7085, BMS-345541, MLN-0415), or even phytochemicals like curcumin, resveratrol or epigallocatechin gallate. Based on the TAM-PA and TAM-PS341 combinations evaluated here, a TAM-sensitizing drug dose affecting a high-risk ER-positive breast cancer would be expected to restore ER-NCoR binding and could be monitored during therapy by tumor reduction in the validated NFκB and AP-1 gene expression signature.

## Conclusion

The findings presented here implicate increased NFκB and AP-1 transcriptional responses in tamoxifen resistant ER-positive breast cancer models. A minimal set of transcripts reflecting NFκB and AP-1 upregulated genes may be able to identify node-negative ER-positive primary breast cancers at risk for early clinical relapse despite tamoxifen therapy. Agents capable of preventing NFκB and AP-1 gene activation may be able to improve the antiestrogen responsiveness of such high-risk ER-positive breast cancers.

## Competing interests

The author(s) declare that they have no competing interests.

## Authors' contributions

YZ designed and carried out all of the cell line studies and participated in drafting the manuscript. CY analyzed all of the expression array data, researched all reported NFKB and AP-1 upregulated gene transcripts, and contributed to the statistical analysis. JWG, KC, and SHD provided all the UCSF breast cancer samples, RNA extracts, and accompanying clinical data. DHM contributed to the UCSF breast cancer case selection and the outcome and statistical analyses. UE and SE-C provided all the Basel cases, transcript and outcome data. CCB conceived the study, participated in the experimental design, coordinated all studies and their analyses, and drafted the final manuscript. All authors read and approved the final manuscript.

## Pre-publication history

The pre-publication history for this paper can be accessed here:



## Figures and Tables

**Table 1:** Treatment interactions between TAM-PS341 and TAM-PA in ER-positive breast cancer models.

**A.**							
		**MCF7**		**MCF7/HER2**		**BT474**	
				
	TAM (500 nM)	+	+	+	+	+	+
	PS341 (nM)	10	25	10	25	10	25

	% of control:						
	Observed (O)	71	60	77	61	48	35
	Expected (E)	62	53	83	72	80	59

	**Ratio: O/E**	**1.2**	**1.1**	0.93*	0.84*	0.59*	0.59*

**B.**							

		**MCF7**		**MCF7/HER2**		**BT474**	
				
	TAM (500 nM)	+	+	+	+	+	+
	Parthenolide (μM)	1	5	1	5	1	5

	% of control:						
	Observed (O)	91	44	85	44	72	8
	Expected (E)	88	42	84	52	81	13

	**Ratio: O/E**	**1.03**	**1.05**	**1.01**	0.85*	0.89*	0.62*

*p < 0.01 for comparison with MCF7; primary data as originally reported (11).

## References

[B1] O'Lone R, Frith MC, Karlsson EK, Hansen U (2004). Genomic targets of nuclear estrogen receptors. Mol Endocrinol.

[B2] Glidewell-Kenney C, Weiss J, Lee E-J, Pillai S, Ishikawa T, Ariazi EA, Jameson JL (2005). ERE-independent ERα target genes differentially expressed in human breast tumors. Mol Cell Endocrinol.

[B3] Benz CC (1998). Transcription factors and breast cancer. Endocrine-Related Cancer.

[B4] Dobrzycka KM, Townson SM, Jiang S, Oesterreich S (2003). Estrogen receptor corepressors--a role in human breast cancer. Endocrine-Related Cancer.

[B5] Lonard DM, O'Malley BW (2003). The expanding cosmos of nuclear receptor coactivators. Cell.

[B6] Keeton EK, Brown M (2005). Cell cycle progression stimulated by tamoxifen-bound estrogen receptor α and promoter-specific effects in breast cancer cells deficient in NCoR and SMRT. Mol Endocrinol.

[B7] Girault I, Lerebours F, Amarir S, Tozlu S, Tubiana-Hulin M, Lidereau R, Bieche I (2003). Expression analysis of estrogen receptor α coregulators in breast carcinoma: evidence that NCOR1 expression is predictive of the response to tamoxifen. Clin Cancer Res.

[B8] Wang LH, Yang XY, Zhang Z, Ping A, Kim H-J, Huang J, Clarke R, Osborne CK, Inman JK, Appella E, Farrar WL (2006). Disruption of estrogen receptor DNA-binding domain and related intramolecular communication restores tamoxifen sensitivity in resistant breast cancer. Cancer Cell.

[B9] Kalaitzidis D, Gilmore TD (2005). Transcription factor cross-talk: the estrogen receptor and NF-kappaB. Trends Endocrinol Metab.

[B10] Biswas DK, Singh S, Shi Q, Pardee AB, Iglehart JD (2005). Crossroads of estrogen receptor and NF-kappaB signaling. Sci STKE.

[B11] Zhou Y, Eppenberger-Castori S, Eppenberger U, Benz CC (2005). The NFκB pathway and endocrine resistant breast cancer. Endocrine-Related Cancer.

[B12] Webb P, Nguyen P, Valentine C, Lopez GN, Kwok GR, McInerney E, Katzenellenbogen BS, Enmark E, Gustafsson JA, Nilsson S, Kushner PJ (1999). The estrogen receptor enhances AP-1 activity by two distinct mechanisms with different requirements for receptor transactivation functions. Mol Endocrinol.

[B13] Kushner PJ, Agard DA, Green GL, Scanlan TS, Shiau AK, Uht RM, Webb P (2000). Estrogen receptor pathways to AP-1. J Steroid Biochem Mol Biol.

[B14] Jakacka M, Ito M, Weiss J, Chien PY, Gehm BD, Jameson JL (2001). Estrogen receptor binding to DNA is not required for its activity through the nonclassical AP1 pathway. J Biol Chem.

[B15] Cheung E, Acevedo ML, Cole PA, Kraus WL (2005). Altered pharmacology and distinct coactivator usage for estrogen receptor-dependent transcription through activating protein-1. Proc. Natl Acad Sci (USA).

[B16] Dumont JP, Bitoni AJ, Wallace CD, Baumann RJ, Cashman EA, Cross-Doersen DE (1996). Progression of MCF-7 breast cancer cells to antiestrogen-resistant phenotype is accompanied by elevated levels of AP-1 DNA-binding activity. Cell Growth and Differentiation.

[B17] Johnston SR, Lu B, Scott GK, Kushner PJ, Smith IE, Dowsett M, Benz CC (1999). Increased activator protein-1 DNA binding and c-Jun NH2-terminal kinase activity in human breast tumors with acquired tamoxifen resistance. Clin Cancer Res.

[B18] Benz CC, Scott GK, Sarup JC, Johnson RM, Tripathy D, Coronado E, Shepard HM, Osborne CK (1992). Estrogen-dependent, tamoxifen-resistant tumorigenic growth of MCF-7 cells transfected with HER2/*neu*. Breast Cancer Res Treat.

[B19] Zhou Y, Eppenberger-Castori S, Marx C, Yau C, Scott GK, Eppenberger U, Benz CC (2005). Activation of nuclear factor-κB (NFκB) identifies a high-risk subset of hormone-dependent breast cancers. Int J Biochem Cell Biol.

[B20] Yau C, Fedele V, Hubbard A, Moore D, Chew K, Dairkee S, Tommasi S, Paradiso A, Gray J, Albertson D, Benz CC (2006). Age-associated differences in primary breast cancer gene expression profiles. Proc.

[B21] Van't Veer LJ, Dai H, van de Vijver MJ, He YD, Hart AA, Mao M, Peterse HS, van der Kooy K, Marton MJ, Witteveen AT, Schreiber GJ, Kerkhoven RM, Roberts C, Linsley PS, Bernards R, Friend SH (2002). Gene expression profiling predicts clinical outcome of breast cancer. Nature.

[B22] Irigoyen JP, Munoz-Canoves P, Montero L, Koziczak M, Nagamine Y (1999). The plasminogen activator system: biology and regulation. Cell Mol Life Sci.

[B23] Sliva D, English D, Lyons D, Lloyd FP Jr (2002). Protein kinase C induces motility of breast cancers by upregulating secretion of urokinase-type plasminogen activator through activation of AP-1 and NF-κB. Biochem Biophys Res Commun.

[B24] Fujioka S, Niu J, Schmidt C, Sclabas GM, Peng B, Uwagawa T, Li Z, Evans DB, Abbruzzese JL, Chiao PJ (2004). NFκB and AP-1 connection: mechanism of NFκB dependent regulation of AP-1 activity. Mol Cell Biol.

[B25] Lewis JA, Thomas TJ, Pestell RG, Albanese C, Gallo MA, Thomas T (2005). Differential effects of 16a-hydroxyestrone and 2-methoxyestradiol on cyclin D1 involving the transcription factor ATF-2 in MCF7 breast cancer cells. J Mol Endo.

[B26] Seeger H, Wallwiener D, Mueck AO (2006). Different effects of estradiol and various antiestrogens on TNF-alpha-induced changes of biochemical markers for growth and invasion of human breast cancer cells. Life Sci.

[B27] Wang Y, KIijn JG, Zhang Y, Sieuwerts AM, Look MP, Yang F, Talantov D, Timmermans M, Meijer-van Gelder ME, Yu J, Jatkoe T, Berns EM, Atkins D, Foekens JA (2005). Gene expression profiles to predict distant metastasis of lymph node-negative primary breast cancer. Lancet.

[B28] Urban P, Vuaroqueaux V, Labuhn M, Delorenzi M, Wirapati P, Wight E, Senn H-J, Benz C, Eppenberger U, Eppenberger-Castori S (2006). Increased expression of urokinase-type plasminogen activator mRNA determines adverse prognosis in ErbB2-positive primary breast cancer. J Clin Oncol.

[B29] Cogswell PC, Gutteridge DC, Funkhouser WK, Baldwin AS (2000). Selective activation of NF-κB subunits in human breast cancer: potential roles for p52 and for Bcl-3. Oncogene.

[B30] Ghosh S, Karin M (2002). Missing pieces in the NF-κB puzzle. Cell.

[B31] Pratt MA, Bishop TE, White D, Yasvinski G, Menard M, Niu MY, Clarke R (2003). Estrogen withdrawal-induced NF-kappaB activity and bcl-3 expression in breast cancer cells: roles in growth and hormone independence. Mol Cell Biol.

[B32] Benz CC, Dowsett M, Ingle J (2004). ErbB2/HER2 and other molecular pathways in ER-positive breast cancer: impact on endocrine resistance and clinical outcome. Endocrine Therapy in Breast Cancer.

[B33] DeLaurentiis M, Arpino G, Massarelli E, Ruggiero A, Carlomagno C, Ciardiello F, Tortora G, D'Agostino D, Caputo F, Cancello G, Montagna E, Malorni L, Zinno L, Lauria R, Bianco AR, De Placido S (2005). A meta-analysis on the interaction between HER-2 expression and response to endocrine treatment in advanced breast cancer. Clin Cancer Res.

[B34] Sorlie T, Perou CM, Tibshirani R, Aas T, Geisler S, Johnson H, Hastie T, Eisen MB, van de Rijn M, Jeffrey SS, Thorsen T, Quist H, Matese JC, Brown PO, Botstein D, Lonning PE, Borresen-Dale A-L (2001). Gene expression patterns of breast carcinomas distinguish tumor subclasses with clinical implications. Proc Natl Acad Sci (USA).

[B35] Gruvberger S, Ringner M, Chen Y, Panavally S, Saal LH, Borg A, Ferno M, Peterson C, Meltzer PS (2001). Estrogen receptor status in breast cancer is associated with remarkably distinct gene expression patterns. Cancer Res.

[B36] Jansen MPHM, Foekens JA, vanStaveren IL, Dirkzwager-Kiel MM, Ritstier K, Look MP, Meijer-van Gelder ME, Sieuwerts AM, Portengen H, Dorssers LCJ, Klijn JGM, Berns EMJJ (2005). Molecular classification of tamoxifen-resistant breast carcinomas by gene expression profiling. J Clin Oncol.

[B37] Kurokawa H, Arteaga CL (2003). ErbB (HER) receptors can abrogate antiestrogen action in human breast cancer by multiple signaling mechanisms. Clin Cancer Res.

[B38] Schiff R, Massarweh SA, Shou J, Bharwani L, Mohsin IK, Osborne CK (2004). Cross-talk between estrogen receptor and growth factor pathways as a molecular target for overcoming endocrine resistance. Clin Cancer Res.

[B39] Shou J, Massarweh S, Osborne CK, Wakeling AE, Åli S, Weiss H, Schiff R (2004). Mechanisms of tamoxifen resistance: increased estrogen receptor-HER2/neu cross-talk in ER/HER2-positive breast cancer. J Natl Cancer Inst.

[B40] Come SE, Buzdar AU, Ingle JN, Arteaga CL, Brown M, Dowsett M, Hilsenbeck SG, Kumar R, Johnston SRD, Lee AV, Paik S, Pritchard KI, Winer EP, Hart C (2006). Proceedings of the fifth international conference on recent advances and future directions in endocrine therapy for breast cancer: conference summary statement. Clin Cancer Res.

[B41] Parisot JP, Hu XF, DeLuise M, Zalcberg JR (1999). Altered expression of the IGF-1 receptor in a tamoxifen-resistant human breast cancer cell line. Br J Cancer.

[B42] Campbell RA, Bhat-Nakshatri P, Patel NM, Constantinidou D, Ali S, Nakshatri H (2001). Phosphatidylinositol 3-kinase/AKT-mediated activation of estrogen receptor alphas: a new model for anti-estrogen resistance. J Biol Chem.

[B43] Chisamore MJ, Ahmed Y, Bentrem DJ, Jordan VC, Tonetti DA (2001). Novel antitumor effect of estradiol in athymic mice injected with a T47D breast cancer cell line overexpressing protein kinase Calpha. Clin Cancer Res.

[B44] Gee JM, Howell A, Gullick WJ, Benz CC, Sutherland RL, Santen RJ, Martin LA, Ciardiello F, Miller WR, Dowsett M, Barrett-Lee P, Robertson JF, Johnston SR, Jones HE, Wakeling AE, Duncan R, Nicholson RI (2005). Consensus statement. Workshop on therapeutic resistance in breast cancer: impact of growth factor signaling pathways and implications for future treatment. Endocrine-Related Cancer.

[B45] Johnston SRD (2006). Clinical efforts to combine endocrine agents with targeted therapies against epidermal growth factor receptor/human epidermal growth factor receptor 2 and mammalian target of rapamycin in breast cancer. Clin Cancer Res.

[B46] Kurokawa H, Lenferink AEG, Simpson JF, Pisacane PI, Sliwkowski MX, Forbes JT, Arteaga CL (2000). Inhibition of HER2/neu (erbB2) and mitogen-activated protein kinases enhances tamoxifen action against HER2-overexpressing, tamoxifen-resistant breast cancer cells. Cancer Res.

[B47] Meijer-van Gelder ME, Look MP, Peters HA, Schmitt M, Brunner N, Harbeck N, Klijn JGM, Foekens JA (2004). Urokinase-type plasminogen activator system in breast cancer: association with tamoxifen therapy in recurrent disease. Cancer Res.

[B48] Butt AJ, McNeil CM, Musgrove EA, Sutherland RL (2005). Downstream targets of growth factor and oestrogen signaling and endocrine resistance: the potential roles of c-Myc, cyclin D1 and cyclin E. Endocrine-Related Cancer.

[B49] Paik S (2006). Methods for gene expression profiling in clinical trials of adjuvant breast cancer therapy. Clin Cancer Res.

[B50] Surh Y-J, Kundu JK, Na H-K, Lee J-S (2005). Redox-sensitive transcription factors as prime targets for chemoprevention with anti-inflammatory and antioxidative phytochemicals. J Nutr.

